# Supplementary figures and images for: Chemical analysis of callus extracts from toxic and non-toxic varieties of Jatropha curcas L
Source: PeerJ. 2020 Nov 11;8:e10172. doi: 10.7717/peerj.10172 (PMC7666564; doi:10.7717/peerj.10172)

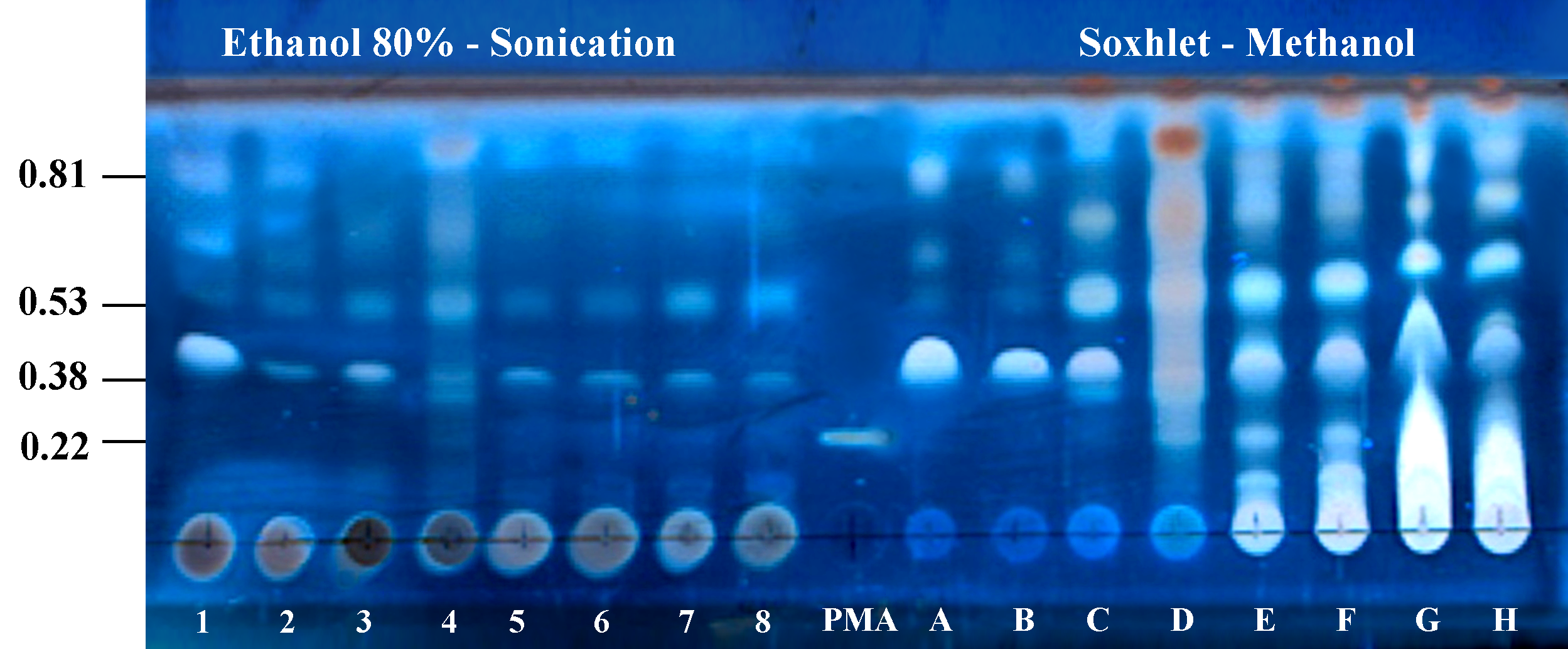

Supplement: Supplemental Information 1 — The extracts obtained with ethanol 80% - sonication are referred with numbers (1 8). The extracts obtained with Soxhlet methanol are referred with letters (A H). PMA: Phorbol-12-myristate-13-acetate Rf 0.22 (Sigma, PE reference standard). Toxic variety seed (1 and A), Non-toxic variety seed (2 and B), Toxic variety leaves (3 and C), Non-toxic variety leaves (4 and D), Toxic variety-callus 14 d (5 and E), Toxic variety-callus 38 d (6 and F), Non-toxic variety-callus 14 d (7 and G), Non-toxic variety-callus 38 d (8 and H). Mobile phase chloroform-methanol (97:3), cerium sulfate-revealed, observed at 366 nm UV light. [file peerj-08-10172-s001.png]

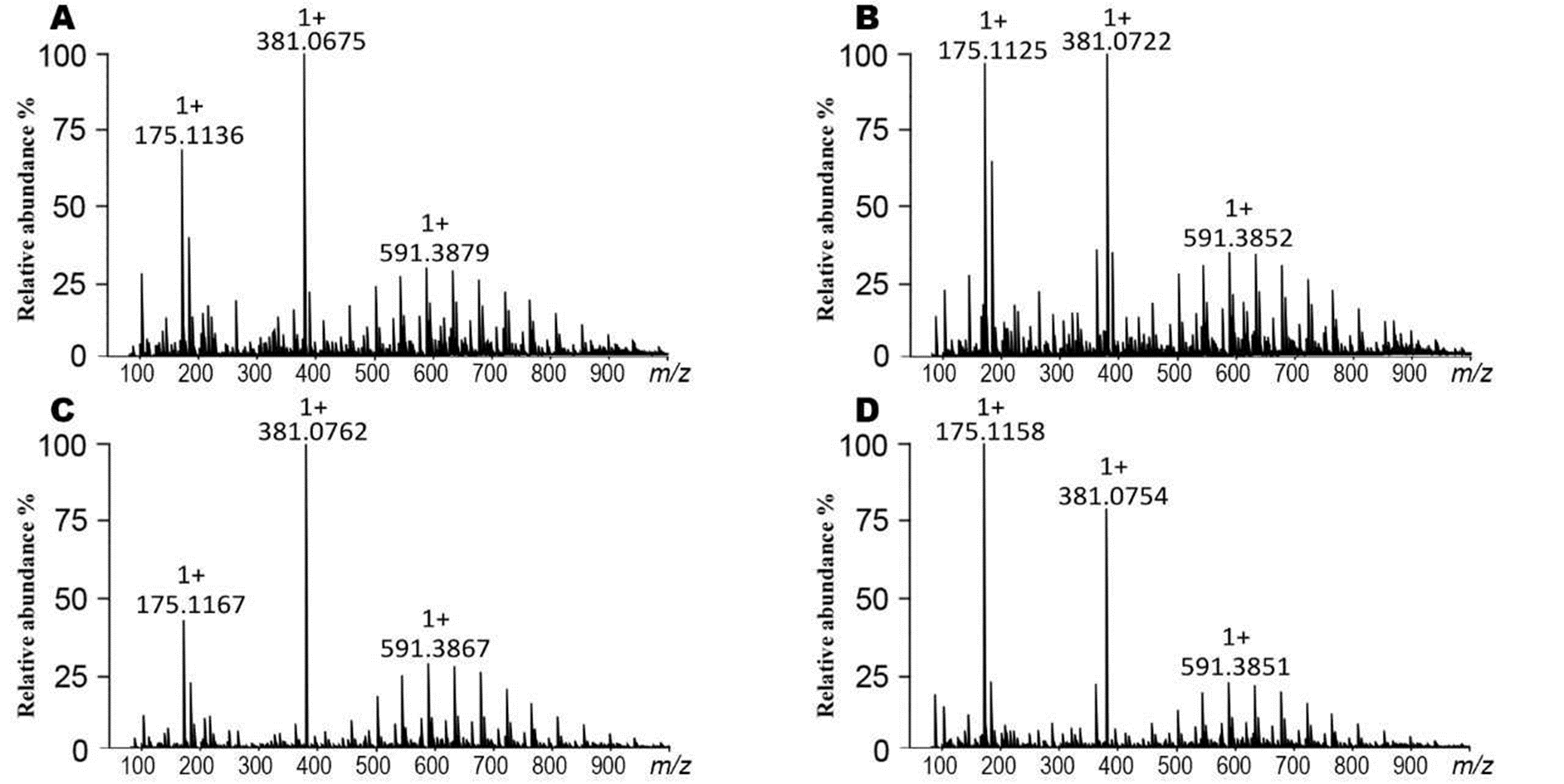

Supplement: Supplemental Information 2 — Toxic variety-callus 14 d extract (A), toxic variety-callus 38 d extract (B), non-toxic variety-callus 14 d extract (C), and non-toxic variety-callus 38 d extract (D). [file peerj-08-10172-s002.png]

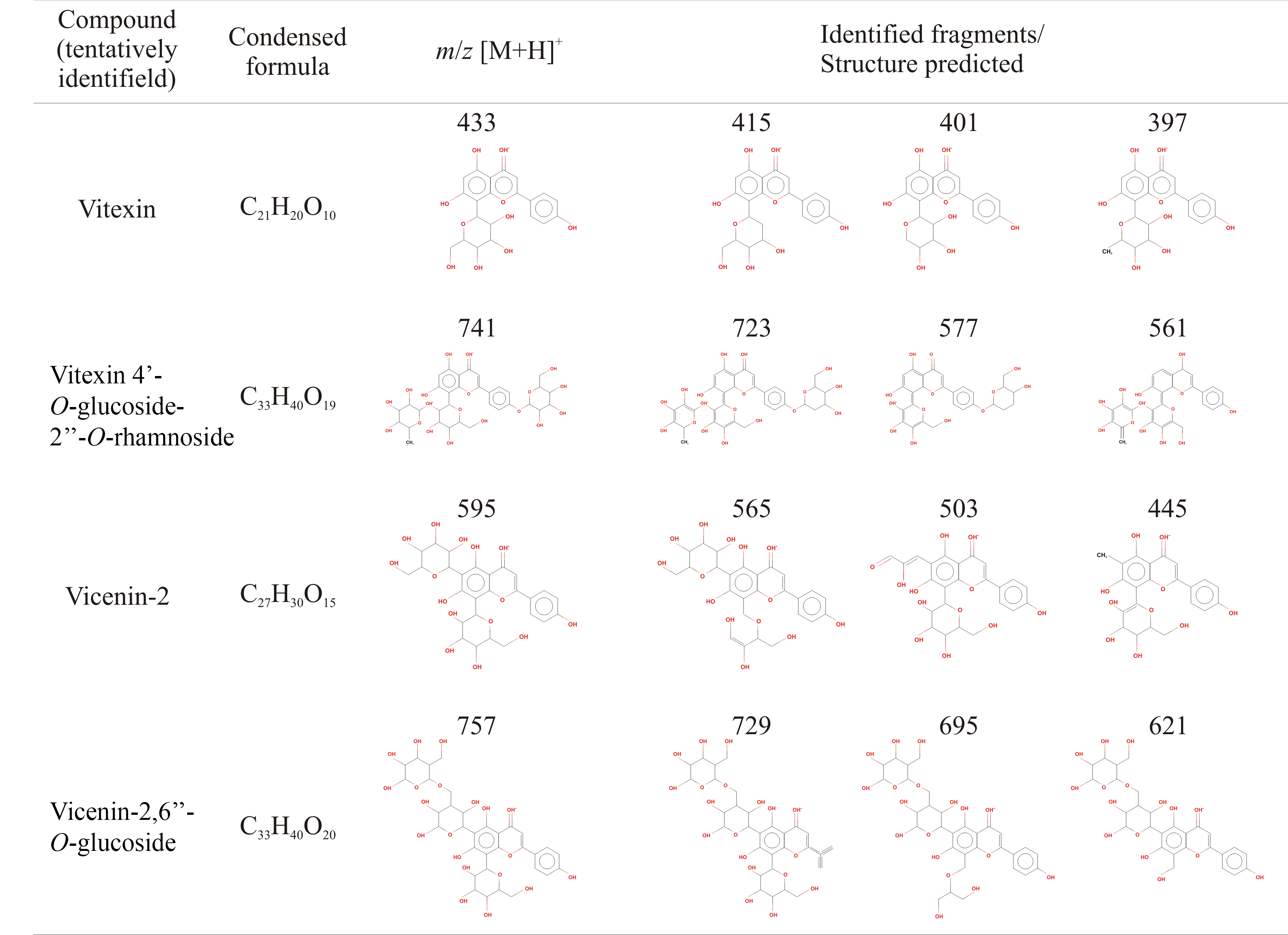

Supplement: Supplemental Information 3 — It is included the predictive structure corresponding to vicenin-2,6”-O-Glucoside m/z 757 [M+H]+ which is not reported to, but it is to Stellaria holostea. [file peerj-08-10172-s003.png]
